# Timing of stroke risk reassessment in atrial fibrillation patients with a CHA_2_DS_2_-VA score of 0 or 1: the Norwegian AFNOR study

**DOI:** 10.1093/europace/euaf145

**Published:** 2025-07-24

**Authors:** Mariam Anjum, Inger Ariansen, Marius Myrstad, Lars J Kjerpeseth, Vidar Hjellvik, Eva Skovlund, Ingrid E Christophersen, Arnljot Tveit, Trygve Berge

**Affiliations:** Department of Medical Research, Bærum Hospital, Vestre Viken Hospital Trust, Gjettum, Norway; Department of Chronic Diseases, Norwegian Institute of Public Health, Oslo, Norway; Department of Internal Medicine, Bærum Hospital, Vestre Viken Hospital Trust, Gjettum, Norway; Faculty of Medicine, Institute of Clinical Medicine, University of Oslo, Oslo, Norway; Department of Chronic Diseases, Norwegian Institute of Public Health, Oslo, Norway; Department of Medical Research, Bærum Hospital, Vestre Viken Hospital Trust, Gjettum, Norway; Department of Internal Medicine, Bærum Hospital, Vestre Viken Hospital Trust, Gjettum, Norway; Department of Chronic Diseases, Norwegian Institute of Public Health, Oslo, Norway; Department of Chronic Diseases, Norwegian Institute of Public Health, Oslo, Norway; Department of Public Health and Nursing, Norwegian University of Science and Technology, Trondheim, Norway; Department of Medical Research, Bærum Hospital, Vestre Viken Hospital Trust, Gjettum, Norway; Department of Medical Genetics, Oslo University Hospital, Oslo, Norway; Department of Medical Research, Bærum Hospital, Vestre Viken Hospital Trust, Gjettum, Norway; Faculty of Medicine, Institute of Clinical Medicine, University of Oslo, Oslo, Norway; Department of Medical Research, Bærum Hospital, Vestre Viken Hospital Trust, Gjettum, Norway

**Keywords:** Atrial fibrillation, Dynamic care, Reassessment, Stroke prevention, Risk factors

## Abstract

**Aims:**

Stroke risk in atrial fibrillation (AF) patients increases over time, but the optimal reassessment interval remains unclear. This study evaluated changes in the CHA_2_DS_2_-VA score in AF patients with low (score 0) or intermediate (score 1) stroke risk and explored appropriate reassessment intervals.

**Methods and results:**

Using Norwegian national registries (2011–18), 40 782 individuals with incident AF aged ≥18 years and a low or intermediate CHA_2_DS_2_-VA score were identified. Patients were followed from first AF diagnosis until an increase in the CHA_2_DS_2_-VA score, and the proportion with increased score was assessed across age groups. The number needed to reassess to detect one new CHA_2_DS_2_-VA risk factor was calculated at different time intervals after AF diagnosis. The CHA_2_DS_2_-VA score increased in 50% of patients after a median follow-up of 1.7 years. The proportion of patients with an increased CHA_2_DS_2_-VA score was 19% at 6 months, 25% at 1 year, and 40% at 3 years after AF diagnosis. At 1 year, the proportion of patients with a new risk factor was lower in those aged 18–44 years (8%) and 45–54 years (14%) compared to those aged >55 years (30%), with the number needed to reassess at 1 year being 12, 7, and 3 patients, respectively.

**Conclusion:**

New risk factors emerged in half of AF patients within 1.7 years. Age-specific differences underscore the need for tailored reassessment, suggesting a shorter interval of 6 months for patients ≥55 years and 1 year for those <55 years and routinely at age 65 and 75 years.

## Graphical Abstract

**Graphical Abstract:**
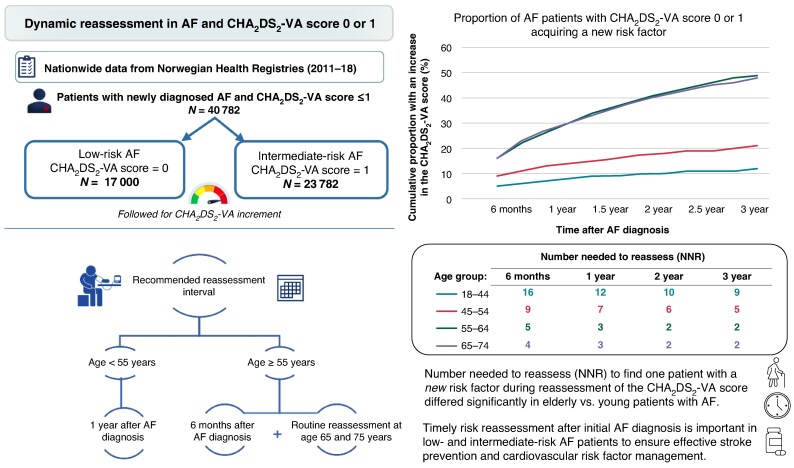

What's new?

- Evidence on how CHA_2_DS_2_-VA score progression varies across different subgroups is lacking, and the optimal time interval of reassessing the cardiovascular risk in atrial fibrillation (AF) patients with a CHA_2_DS_2_-VA score of 0 or 1 remains unclear.
- This is the first large real-world study investigating the optimal frequency of stroke risk reassessment in low- and intermediate-risk AF patients across different age groups and the first large-scale study exploring risk factor reassessment in a European population.
- Half of the patients developed a new risk factor over a median follow-up of 1.7 years. Proportion of patients with increased CHA_2_DS_2_-VA score 1 year after AF diagnosis was 8% in the youngest group and 30% in the oldest group.
- Timely reassessment is important in AF. Age-specific variations in stroke risk progression highlight the importance of tailored reassessment intervals, suggesting a shorter interval of 6 months in older (≥55 years) AF patients and 1 year in younger patients (<55 years).

## Introduction

Patients with atrial fibrillation (AF) may have increased risk of stroke, but the risk varies according to age and additional risk factors. The European Society of Cardiology (ESC) 2024 guidelines for the management of AF recommend the CHA_2_DS_2_-VA score as a tool for estimating the individual stroke risk in AF patients. The sex category was recently removed from the CHA_2_DS_2_-VASc score, yielding the CHA_2_DS_2_-VA score, which has demonstrated similar predictive accuracy for stroke risk in AF patients.^1,2^ In most studies, the CHA_2_DS_2_-VA score is calculated only once, based on baseline risk factors, whereas the outcomes are assessed over a follow-up period of 1–10 years.^3^ European guidelines strongly recommend oral anticoagulants (OACs) for high-risk patients (CHA_2_DS_2_-VA score ≥ 2), highlighting the positive net clinical benefit when weighing stroke risk against bleeding risk. Meanwhile, the guidelines advise against OACs in low-risk patients with a CHA_2_DS_2_-VA score of 0 due to an increased risk of bleeding but suggest that OACs should be considered for intermediate-risk patients with a CHA_2_DS_2_-VA score of 1.^1^ However, the CHA_2_DS_2_-VA score is not static. While age increases progressively over time, other risk factors, such as hypertension, diabetes mellitus, vascular disease, and deterioration of other conditions leading to congestive heart failure or stroke, may occur incidentally.

Studies in Asian populations suggest that updated CHA_2_DS_2_-VASc scores and changes over time (delta CHA_2_DS_2_-VASc) are valuable predictors of ischaemic stroke.^4,5^ Given the dynamic nature of cardiovascular risk factors, their assessment and management in patients with AF should also be dynamic. Early identification and treatment of cardiovascular risk factors are crucial for slowing AF progression, improving the quality of life, and preventing adverse outcomes, such as heart failure or stroke.^6,7^ Moreover, reassessment may lead to timely initiation of OAC treatment, ultimately reducing the risk of stroke and death, but the optimal interval for reassessment remains unclear.

Recent updates in the 2024 ESC/European Association for Cardio-Thoracic Surgery guidelines include the introduction to the AF-CARE framework, which recognizes the need to repeated evaluation and adjustment while managing patients with AF. The ‘E’ pillar of the AF-CARE represents ‘Evaluation and dynamic reassessment’, emphasizing the importance of risk reassessment over time.^1,8^

European Society of Cardiology 2024 guidelines recommend clinical reassessment 6 months after initial presentation with AF and then at least annually, whereas the American and Australian guidelines advocate for annual reassessment.^1,9,10^ The Asian Pacific Hearth Rhythm Society (APHRS) advices reassessment every 4 months if possible and at least annually.^11^

Atrial fibrillation patients represent a heterogeneous population, from young, otherwise healthy individuals who have good rhythm control and infrequent AF paroxysms to older individuals with a higher burden of cardiovascular risk factors and comorbidities. Stroke prevention in AF continues to evolve, with recent literature emphasizing a more holistic, dynamic, and individualized approach to patient management.^12,13^ Current guidelines recommend the same cardiovascular risk reassessment for all AF patients; however, the optimal time for reassessment may vary across subgroups, warranting tailored reassessment that considers risk trajectories. Furthermore, there is a lack of evidence to define the most appropriate reassessment interval for European AF populations and across age groups.

This study aimed to investigate the incidence of cardiovascular risk factors included in the CHA_2_DS_2_-VA score and to quantify the incidence of CHA_2_DS_2_-VA score increment in patients with AF and low or intermediate stroke risk. Furthermore, we aimed to explore the optimal time interval for reassessing cardiovascular risk factors in AF patients with low and intermediate stroke risk, as well as subgroup analyses across sex and age. Lastly, we aimed to calculate the number of patients that would have to be reassessed to identify one patient with a newly acquired CHA_2_DS_2_-VA risk factor at different time intervals in a real-world AF population.

## Methods

### Study design and data sources

The Atrial Fibrillation in Norway (AFNOR) study is a nationwide cohort study based on linked individual level health data from population-based registries in Norway, including the Norwegian Patient Registry, the Norwegian Prescription Database (NorPD), the Norwegian Cause of Death Registry, and the Population Registry. The Norwegian Patient Registry holds primary and secondary hospital discharge diagnoses codes for both in- and outpatient visits, according to the International Classification of Diseases, 10th Revision (ICD-10). The NorPD includes all prescription drugs dispensed at Norwegian pharmacies since 2004, with detailed information such as Anatomical Therapeutic Chemical classification, brand, strength, pack size, and reimbursement diagnosis codes based on ICD-10 or the second version of the International Classification of Primary Care (ICPC-2).^14^

### Study population

**Figure 1:**
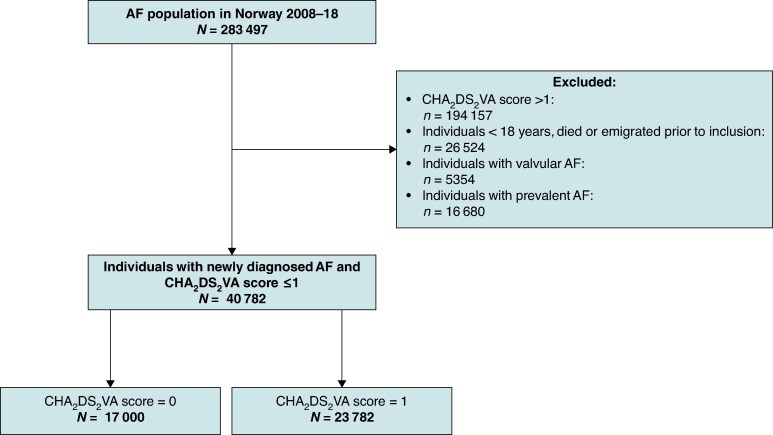
Inclusion flow chart: included patients were followed until increase in the CHA_2_DS_2_-VA score, emigration, mortality, or study end 31 December 2018. AF, atrial fibrillation.

The study population (*n* = 40 782) comprised all Norwegian citizens ≥18 years diagnosed with incident AF and a CHA_2_DS_2_-VA score of 0 or 1 (*Figure 1*; Supplementary material online, *Figure S1*). Individuals were followed from the date of registration of their first AF diagnosis, from 1 January 2011 (study entry), until they developed a new CHA_2_DS_2_-VA risk factor, died, emigrated, or reached the end of study period (31 December 2018). Individuals diagnosed with incident AF during the study period entered the cohort at the time of their AF diagnosis. Individuals with prevalent AF at the baseline, mitral stenosis, or mechanical heart valves were excluded, with the purpose to establish a cohort of patients with newly diagnosed non-valvular AF at low or intermediate stroke risk. Low-risk patients were not included in further analyses as intermediate-risk patients because they would no longer be classified as *newly diagnosed* AF patients at the time their CHA_2_DS_2_-VA score increased from 0 to ≥1.

### Definition of atrial fibrillation and comorbid conditions

Atrial fibrillation and baseline comorbidities were defined using diagnoses registered prior to cohort entry, with a minimum look-back period of 3 years, and successively during follow-up. Diagnosis codes from prescription reimbursement in the NorPD, in- and outpatient contacts in the Norwegian Patient Registry, and the Norwegian Cause of Death Registry were used to define AF and comorbidities. The CHA_2_DS_2_-VA score was calculated based on age, diagnosis codes, and prescription claims and was regarded as a time-varying, dynamic variable. Detailed information on the registries, including the diagnosis and reimbursement codes used to define AF and comorbidities, is available in the online Supplementary material.

### Outcomes

The main outcome of interest was the proportion of patients with an increase in the CHA_2_DS_2_-VA score from 0 to ≥1 in low-risk patients with AF and from 1 to ≥2 in intermediate-risk patients with AF. We also assessed the risk of developing each CHA_2_DS_2_-VA risk factor separately and estimated the time from initial AF diagnosis to the occurrence of a new risk factor. The number needed to reassess (NNR) to identify one patient with a new CHA_2_DS_2_-VA risk factor was calculated across different age groups with the aim to estimate the number of patients that would have to be reassessed at specific time intervals after the AF diagnosis to identify one patient with an increase in their risk of stroke.

### Statistical analysis

Baseline characteristics of the study population are presented as means (standard deviations) for continuous variables and proportions (percentages) for categorical variables. Incidence rates of CHA_2_DS_2_-VA score increment was calculated as the number of events per 100 person-years (%/py) with 95% confidence intervals (CIs). We performed subgroup analyses stratified by sex and age in low- and intermediate-risk patients, using the following age groups: 18–44, 45–54, 55–64, and 65–74 years. The Kaplan–Meier method was used to estimate cumulative incidence curves for newly acquired risk factors. The NNR was calculated as the inverse of the absolute risk at different time intervals following an AF diagnosis as [NNR = 1/(*n*/*N*_0_), where *n* is the number of individuals with a new risk factor and *N*_0_ is the total number of individuals in the relevant group]. In simpler terms, NNR reflects the number of patients that need to be reassessed to identify one individual with a new risk factor. For example, if 10% of patients develop a new risk factor within 1 year, the NNR would be 1/0.10, meaning 10 patients would need to be reassessed to detect one new risk factor. We performed analyses for the full study period, with particular focus on the initial time intervals up to 3 years. The analyses were performed in the Stata (version 18) statistical software.

### Ethical approval

There was no direct patient involvement in this research. The study complied with all legal and regulatory requirements and was approved by the Norwegian Regional Committee for Medical and Health Research Ethics (Ref 12315/2020), Region South-East, and has an approved Data Privacy Impact Assessment.

## Results

### Baseline characteristics

In total, 40 782 patients with incident AF (33% women) and a CHA_2_DS_2_-VA score of 0 or 1 were included (*Figure 1*). Among these, 17 000 (41.7%) had low stroke risk (CHA_2_DS_2_-VA score 0) and 23 782 (58.3%) had intermediate stroke risk (CHA_2_DS_2_-VA score 1) (*Table 1*). The mean age was 53.1 years (SD 9.7) in the low-risk group and 64.8 years (SD 8.2) in the intermediate-risk group. Among intermediate-risk patients, the most common baseline risk factor was age 65–74 years (54.2%), followed by hypertension (26.0%), vascular disease (5.5%), heart failure (5.4%), and diabetes (3.9%). Sex-stratified clinical characteristics are provided in Supplementary material online, *Table S1*.

**Table 1:** Baseline characteristics

	Low-risk CHA_2_DS_2_-VA score 0	Intermediate-risk CHA_2_DS_2_-VA score 1	All low + intermediate risk
*n* (%)	17.000 (41.7)	23.782 (58.3)	40.782 (100)
Female sex (%)	5.015	8.545	13.469 (33)
Men (%)	11.985	15.328	27.213 (67)
Mean age. years (SD)	53.1 (9.7)	64.8 (8.2)	59.9 (10.5)
Distribution of age, *n* (%)			
Age 18–44 (%)	3.433 (20.2)	747 (3.1)	4.180 (10.3)
Age 45–54 (%)	4.670 (27.5)	2.137 (9.0)	6.807 (16.7)
Age 55–64 (%)	8.897 (52.3)	6.715 (28.2)	15.612 (38.3)
Age 65–74 (%)		14.183 (59.6)	14.183 (35.8)
CHA_2_DS_2_-VASc risk factors			
Heart failure (%)		1.287 (5.4)	1.287 (3.2)
Hypertension (%)		6.173 (26.0)	6.173 (15.1)
Age 65–74 (%)		14.081 (59.2)	14.081 (34.6)
Diabetes (%)		933 (3.9)	933 (2.3)
Vascular disease (%)		1.308 (5.5)	1.308 (3.2)
Abnormal liver function (%)	202 (1.2)	422 (1.8)	624 (1.5)
Anaemia (%)	755 (4.4)	1.642 (6.9)	2.397 (5.9)
Alcohol misuse (%)	546 (3.2)	806 (3.4)	1.352 (3.3)
Cancer (%)	1.150 (6.8)	3.030 (12.7)	4.180 (10.3)
Renal disease (%)	329 (1.9)	1.096 (4.6)	1.425 (3.5)

### Changes in the CHA_2_DS_2_-VA score

During a median follow-up time of 1.74 years [interquartile range (IQR) 0.5–3.8], the CHA_2_DS_2_-VA score increased in 50% (95% CI 49.5–50.5) of the patients with low or intermediate stroke. The average annual incidence rate of acquiring at least one new CHA_2_DS_2_-VA risk factor was 21% (see Supplementary material online, *Table S2*).

Among low-risk patients, 43% (95% CI 42.4–43.9) developed at least one CHA_2_DS_2_-VA risk factor over a median follow-up time of 2.2 years (IQR 0.7–4.4), whereas 55% (95% CI 54.3–55.5) of intermediate-risk patients attained a new risk factor during a median follow-up of 1.5 years (IQR 0.5–3.2). The average annual incidence rates of increased CHA_2_DS_2_-VA score were 15.8% for low-risk patients and 26.2% for intermediate-risk patients.

### Time to risk factor development

Following AF diagnosis, the cumulative proportion of patients with a CHA_2_DS_2_-VA score increment was 19% at 6 months, 25% at 1 year, 34% at 2 years, and 40% at 3 years (*Table 2*). The difference in risk factor attainment between 4 and 6 months following AF diagnosis was minor, with the NNR showing a decrease from 6 at 4 months to 5 at 6 months. At 1 year after AF diagnosis, the overall NNR was 4 with a small difference between intermediate- and low-risk patients (NNR = 4 in intermediate-risk patients; NNR = 5 in low-risk patients).

**Table 2:** Proportion of patients with an increase in the CHA_2_DS_2_-VA score and number needed to reassess at different time intervals after atrial fibrillation diagnosis by the CHA_2_DS_2_-VA risk group, stratified by age with 95% confidence interval

	All Low + intermediate risk		Low-risk CHA_2_DS_2_-VA score 0		Intermediate-risk CHA_2_DS_2_-VA score 1	
	Proportion	NNR	Proportion	NNR	Proportion	NNR
4 months						
All	15.7 (15.4–16.1)	6 (6–7)	13.7 (13.2–14.2)	7 (7–8)	17.2 (16.7–17.7)	6 (6–6)
18–44	5.3 (4.6–6.0)	19 (17–22)	5.0 (4.3–5.8)	20 (17–23)	6.6 (5.0–8.6)	15 (12–20)
45–54	9.7 (9.0–10.4)	10 (10–11)	10.1 (9.3–11.0)	10 (9–11)	8.8 (7.6–10.0)	11 (10–13)
55–64	18.2 (17.6–18.8)	6 (5–6)	18.9 (18.1–19.8)	5 (5–6)	17.1 (16.3–18.1)	6 (6–6)
65–74	19.1 (18.4–19.7)	5 (5–5)			19.1 (18.4–19.7)	5 (5–5)
6 months						
All	19 (18.6–19.4)	5 (5–5)	16.5 (15.9–17)	6 (6–6)	20.8 (20.3–21.3)	5 (5–5)
18–44	6.3 (5.6–7.1)	16 (14–18)	6.0 (5.3- 6.9)	17 (15–19)	7.5 (5.8–9.6)	13 (10–17)
45–54	11.2 (10.5–12.0)	9 (8–10)	11.7 (10.8- 12.6)	9 (8–9)	10.4 (9.1–11.7)	10 (9–11)
55–64	22.0 (21.4–22.7)	5 (4–5)	23.0 (22.1- 23.9)	4 (4–5)	20.7 (19.8–21.7)	5 (5–5)
65–74	23.1 (22.4–23.8)	4 (4–4)			23.1 (22.4–23.8)	4 (4–4)
1 year						
All	25.3 (24.9–25.7)	4 (4–4)	21.6 (21–22.3)	5 (4–5)	27.9 (27.4–28.5)	4 (4–4)
18–44	8.1 (7.3–8.9)	12 (11–14)	7.6 (6.8–8.6)	13 (12–15)	10.0 (8.1–12.4)	10 (8–12)
45–54	14.1 (13.2–14.9)	7 (7–8)	14.4 (13.5–15.5)	7 (6–7)	13.2 (11.8–14.7)	8 (7–8)
55–64	30.2 (29.5–30.9)	3 (3–3)	30.8 (29.8–31.8)	3 (3–3)	29.3 (28.3–30.4)	3 (3–4)
65–74	30.4 (29.7–31.2)	3 (3–3)			30.4 (29.7–31.2)	3 (3–3)
2 years						
All	34.2 (33.8–34.7)	3 (3–3)	29.2 (28.5–29.9)	3 (3–4)	37.8 (37.2–38.4)	3 (3–3)
18–44	10.2 (9.3–11.2)	10 (9–11)	9.7 (8.7–10.7)	10 (9–11)	12.7 (10.5–15.3)	8 (7–10)
45–54	18.2 (17.3–19.1)	6 (5–6)	18.8 (17.7–19.9)	5 (5–6)	16.9 (15.4–18.6)	6 (5–7)
55–64	41.6 (40.8–42.4)	2 (2–2)	42.2 (41.2–43.3)	2 (2–2)	40.8 (39.6–42.0)	2 (2–3)
65–74	40.9 (40.1–41.7)	2 (2–2)			40.9 (40.1–41.7)	2 (2–2)
3 years						
All	40.1 (39.7–40.6)	2 (2–3)	34.1 (33.4–34.9)	3 (3–3)	44.4 (43.8–45)	2 (2–2)
18–44	11.8 (10.8–12.8)	9 (8–9)	11.2 (10.2–12.3)	9 (8–10)	14.2 (11.9–16.9)	7 (6–8)
45–54	20.7 (19.7–21.6)	5 (5–5)	21.1 (19.9–22.3)	5 (4–5)	19.8 (18.1–21.5)	5 (5–6)
55–64	49.2 (48.4–50.0)	2 (2–2)	49.8 (48.8–50.9)	2 (2–2)	48.4 (47.2–49.6)	2 (2–2)
65–74	47.8 (47.0–48.6)	2 (2–2)			47.8 (47.0–48.6)	2 (2–2)

Proportion of patients and NNR with 95% confidence intervals (95% CI). NNR refers to the number needed to reassess to find one patient with an increased score at different time intervals after initial presentation of AF. The NNR was calculated as the inverse of the absolute risk [NNR = 1/(*n*/*N*_0_) where *n* is the number with a new risk factor and *N*_0_ is the total study population in the relevant group].

**Figure 2:**
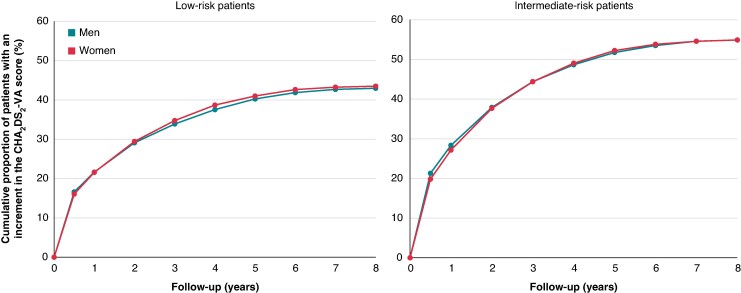
The accumulated frequency of patients with an increase in the CHA_2_DS_2_-VA score during follow-up.

At 6 months after the initial AF diagnosis, 16.5% of low-risk patients and 20.8% of intermediate-risk patients had attained a new risk factor, whereas at 1 year, the proportions increased to 21.6% for low-risk patients and 27.9% for intermediate-risk patients (*Figure 2*).

**Figure 3:**
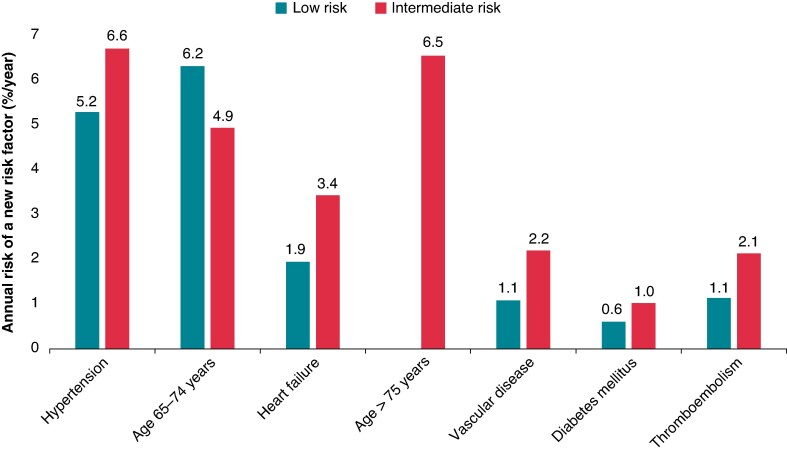
Annual risk (%/per year) of attaining new CHA_2_DS_2_-VA risk factors in low- and intermediate-risk atrial fibrillation patients.

The average annual incidence rate of attaining each of the CHA_2_DS_2_-VA risk factors in the total study population (low- and intermediate-risk patients combined) was 6.0% for hypertension, 5.5% for age 65–74 years, 3.4% for age ≥75 years, 2.7% for heart failure, 1.6% for vascular disease, 1.6% for thromboembolism, and 0.8% for diabetes mellitus (see Supplementary material online, *Table S3*). During an average follow-up of 1 year, 6.3% of low-risk patients reached 65 years of age and 5.2% developed hypertension, while in the intermediate-risk group, 6.5% reached 75 years of age, and 6.6% developed hypertension (*Figure 3*).

**Figure 4:**
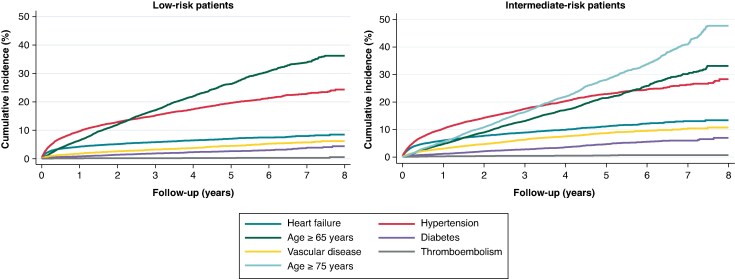
Cumulative incidence of each risk factor component from atrial fibrillation diagnosis up to 8 years of follow-up in low-risk patients (left) and intermediate-risk patients (right).

Age 65–74 years was the most commonly acquired risk factor in low-risk patients overall, although hypertension was more frequent during the first 2 years after AF diagnosis. For intermediate-risk patients, hypertension remained the primary risk factor for the first 3 years before being surpassed by age ≥ 75 years (*Figure 4*).

### Age differences

At 6 months, the proportion with increased CHA_2_DS_2_-VA score was 6.3, 11.0, 22.0, and 23.1% in the age groups 18–44, 45–54, 55–64, and 65–74 years, respectively (see Supplementary material online, *Table S4*). At 1 year, the proportions increased to 8.1, 14.1, 30.2, and 30.4%, respectively. Similarly, the NNR decreased at 1 year, with the lowest values observed in patients aged >55 years of age (NNR = 3; 95% CI 3–3). Stratification by the baseline CHA_2_DS_2_-VA score revealed similar trends.

For low-risk patients, the proportion with an increased CHA_2_DS_2_-VA score at 6 months was 6.0% among those aged 18–44 years, 11.7% in the age group 45–54 years, and 23.0% in the age group 55–64 years, with these proportions rising to 7.6, 14.4, and 30.8% at 1 year after AF diagnosis, respectively.

In intermediate-risk patients, the proportion with a CHA_2_DS_2_-VA increment at 6 months was 7.5, 10.4, 20.7, and 23.1% in those aged 18–44, 45–54, 55–64, and 65–74 years, respectively, increasing to 10.0, 13.2, 29.3, and 30.4%, respectively, 1 year after AF diagnosis.

### Sex differences

Overall, 49.7% of men and 50.7% of women at low or intermediate risk of stroke obtained an increment in the CHA_2_DS_2_-VA score during the study period. Among low-risk patients, 44% of women and 43% of men acquired at least one new stroke risk factor, while among intermediate-risk patients, 55% of both women and men acquired at least one new risk factor. No significant sex differences were observed at any time points assessed (see Supplementary material online, *Table S5*). The NNR is not reported separately by sex since there were no significant differences in the cumulative risk of attaining an increased score.

There were no significant sex differences in the annual risk of acquiring *any* new risk factor, although men, both with low and intermediate stroke risk, had a higher incidence of attaining vascular disease as a risk factor compared to women (see Supplementary material online, *Table S3*). Low-risk women had a higher incidence of attaining age 65–74 years as the first risk factor. In intermediate-risk patients, age ≥75 years and hypertension were risk factors more commonly attained in women, whereas heart failure, diabetes, and vascular disease were more common in men compared to women.

## Discussion

In this nationwide cohort study of more than 40 000 newly diagnosed AF patients with low or intermediate stroke risk (CHA_2_DS_2_-VA score of 0 or 1), we found that 19% of the patients had acquired a new risk factor at 6 months after the diagnosis of AF, increasing to 25% at 1 year. Age was a key determinant of the proportion acquiring new risk factors, with CHA_2_DS_2_-VA score progression varying significantly across age groups. Among those aged 18–44 years, only 8% had a CHA_2_DS_2_-VA score increment within 1 year, compared to 30% of those aged 65–74 years. The NNR to identify one patient with a new risk factor at 6 months ranged from 4 in the oldest to 16 in the youngest age group (*Graphical Abstract*), highlighting the significance of age-specific intervals for clinical reassessment and suggesting closer monitoring of cardiovascular risk factors in older patients with incident AF, who experience more rapid risk progression. In contrast, we did not find any differences in terms of CHA_2_DS_2_-VA score increment between women and men. Atrial fibrillation patients at intermediate stroke risk experienced a modestly higher incidence rate of new risk factors compared to those at low risk.

As far as we know, this is the first large real-world study investigating the optimal frequency of stroke risk reassessment in low- and intermediate-risk AF patients across different age groups, as well as the first large-scale study exploring risk factor reassessment in a non-Asian population.

Previous studies have revealed variations in both time and magnitude of stroke risk progression in individuals with AF. In a cohort study from Taiwan, Chao *et al.*^4^ found that 48.5% of low-risk patients acquired at least one new stroke risk factor during 4 years of follow-up and 80% of these were evident already at 4.2 months after AF diagnosis. Among patients who sustained an ischaemic stroke, 80% showed changes in their stroke risk profile ∼4 months before the event. Based on this, the authors recommended reassessing stroke risk in low-risk patients approximately every 4 months. We did not perform such analysis, as this may introduce biases associated with ‘conditioning on the future’, i.e. interpreting changes occurring after a particular outcome, as this approach may obscure the temporal relationship between risk factor progression and clinical outcomes. Possible explanations for the differing results may be varying methods for baseline risk factor assessment and differences in the rate of follow-up or coding practice. Therefore, the optimal time interval for reassessment of risk factors in individuals with AF may differ across healthcare systems. Of note, the mean age in the Taiwanese cohort was lower than in ours (47.8 years vs. 52.1), suggesting that there may be a faster progression of certain risk factors in the Taiwanese population. Chao *et al.*^15^ included only Asian patients, whose stroke risk and cardiovascular risk profile differ from European populations. Notably, age progression was the most common new risk factor in our cohort, while Chao *et al.* identified hypertension as predominant. These differences highlight the importance of tailoring guideline recommendations to regional contexts and patient demographics. Similarly to Chao *et al.*, we found hypertension to be the most frequently acquired risk factor during the first 2 years after AF diagnosis. With the recent update to European guidelines lowering the thresholds for elevated blood pressure, future studies are needed to assess whether these changes will impact stroke risk in AF patients.^16^

There are few European studies investigating dynamic changes in the risk of stroke in patients with AF and CHA_2_DS_2_-VA score of 0 or 1. Previous studies from Taiwan,^15^ South Korea,^5^ and France^17^ have demonstrated that the CHA_2_DS_2_-VASc score continuously increases over time and that the predictive value of delta CHA_2_DS_2_-VASc score during follow-up was better than the baseline CHA_2_DS_2_-VASc score for prediction of ischaemic stroke.^15^ These studies focused on broader populations including high-risk groups. Similar findings were reported in a *post hoc* analysis of the Motivational Interviewing to Support Oral Anti Coagulation adherence in patients with Atrial Fibrillation trial conducted in Greece.^18^ Our study adds novel insights by focusing specifically on low- and intermediate-risk populations, emphasizing age-specific patterns of risk progression.

In a small Dutch study, the authors compared 41 AF patients with no CHA_2_DS_2_-VA risk factors to healthy patients with sinus rhythm and found that more than half of the AF patients developed a cardiovascular condition during 10 years of follow-up.^19^ The average time to develop a CHA_2_DS_2_-VAsc score ≥ 1 was 4 years. Based on this, the authors recommended 2- to 3-year interval for reassessment of stroke risk. Time to an increased CHA_2_DS_2_-VAsc score in the Dutch study is much longer than what we and other studies have found, which may be due to a small and selected population.

Current AF guidelines recommend periodic reassessment, with the ESC suggesting reassessment 6 months after the initial presentation and annually thereafter, whereas the 2021 APHRS recommends reassessment every 4 months if possible and at least annually.^1,11^ Stroke is a costly healthcare outcome, with long-term costs that often outweigh the price of prevention.^20^ Early detection and timely intervention of cardiovascular risk factors could potentially prevent costly complications, such as stroke or heart failure, thereby improving overall healthcare efficiency. Although frequent monitoring may benefit all patients, limited healthcare resources in both high- and low-income healthcare settings necessitate prioritization based on risk stratification. Tailoring reassessment intervals to patient age, as suggested by our findings, may further optimize AF care and ensure that older patients who are at higher risk of rapid progression are monitored more closely, while the younger can benefit from less frequent assessment without compromising safety.

At all studied time intervals, we found that the NNR was considerably lower in AF patients aged ≥55 years, compared to younger patients. Although the optimal interval for reassessment may vary depending on preferences and available resources, we suggest that reassessment 6 months after a new AF diagnosis is appropriate for those aged ≥55 years (NNR = 5 at 6 months). While the NNR remains higher than 5 for time intervals up to 3 years after the AF diagnoses in patients < 55 years, we believe that the importance of early detection of risk factors justifies accepting a higher NNR in the young and therefore suggest reassessment after 1 year in AF patients younger than 55 years. Atrial fibrillation patients at intermediate stroke risk showed only a slightly higher incidence of new risk factors compared to those at low risk (NNR = 4 vs. NNR = 5). Given this minor difference, we suggest applying the same reassessment strategy in both groups to maintain clinical practicality.

Moreover, we found that transition to a higher age-related risk category (≥65 and ≥75 years) accounted for most of the CHA_2_DS_2_-VA score increments. Age is the most significant risk factor for stroke in AF patients, also in low- and intermediate-risk patients.^21,22^ Although the stroke risk increases continuously with increasing age, the thresholds of 65 and 75 years may serve as clinically relevant minimum of time points where reassessment should be performed at least once. Nevertheless, some Asian studies have demonstrated that the age threshold of 65 years may be too high in Asian patients and have suggested that the age threshold should be reduced to 50 or 55 years, underscoring that age-related risk thresholds may differ across ethnic groups.^23^ Our suggestions are based on the findings of our study, and to the best of our knowledge, no previous studies have investigated age-related differences in stroke risk progression. Further studies are needed to investigate whether the observed trends remain consistent in other populations. Nevertheless, dynamic risk reassessment remains vital for improving patient outcomes, beyond stroke prevention. Early diagnosis and management of comorbidities, such as hypertension, coronary heart disease, and diabetes, can slow down AF progression, improve quality of life, and reduce healthcare burden. Future research should investigate improved surveillance strategies for individuals at low or intermediate risk of stroke, with regular reassessment and improved patient education pertaining to AF-related comorbidity and risk factor management. Incorporation of emerging digital health solutions may further facilitate more personalized and efficient follow-up strategies.

### Limitations

This study has certain limitations. As a register-based cohort study, it is susceptible to misclassification due to potential coding errors. Certain risk factors, such as hypertension and diabetes, are often diagnosed and managed in the primary care, which may lead to underestimation of the CHA_2_DS_2_-VA score in registry-based studies. However, most patients with these diagnoses require pharmacological treatment, which will be captured by the NorPD. Recent data suggest that diabetes prevalence is comparable between the NorPD and primary care registries.^24^ The validity of AF diagnosis and the risk factors used to calculate the CHA_2_DS_2_-VA score in this study are reported to be high in Norwegian registries.^24–28^ There is a risk of underestimating both the baseline and follow-up CHA_2_DS_2_-VA score, due to possibly missed clinical assessments or poor coding practice. The diagnosis of incident AF may lead to increased healthcare contact and diagnostic evaluation, which may result in more intensive detection of comorbidities. This may have resulted in the identification of risk factors that may have been present but previously unrecognized or untreated. Some patients with prevalent AF may have been included, due to the limited look-back period. Moreover, our findings reflect a Northern European study population, which may limit the generalizability to other populations. It was beyond the scope of this study to investigate the use of OAC treatment during follow-up or the incidence of stroke or other cardiovascular endpoints. Although the CHA_2_DS_2_-VA score is widely used to predict stroke risk, it has a moderate predictive accuracy (*C*-statistic ∼ 0.6).^29^ Therefore, it should be recognized as a tool to guide risk assessment rather than a definitive determinant for OAC treatment. Individual clinical evaluation is required to fully assess both the thromboembolic and bleeding risk. We suggest that our findings may be used to support closer monitoring and individualized treatment decisions. This study focuses solely on the CHA₂S₂-VA risk factors; nonetheless, lifestyle changes such as smoking cessation, weight management, and physical activity influence the trajectory of stroke risk and should be part of a reassessment.

One important strength of our study is that it reflects a real-world, contemporary population of individuals with incident AF and low or intermediate risk of stroke, followed over a long period of time with virtually no loss to follow-up. The large sample size reduces the risk of a random error and fully assesses the dynamic nature of the CHA_2_DS_2_-VA score in patients with AF at low and intermediate risk of stroke.

## Conclusion

In this nationwide study, half of the patients with newly diagnosed AF and low or intermediate stroke risk developed a new risk factor during a median follow-up of 1.7 years, with 25% of the patients experiencing a CHA_2_DS_2_-VA score increment within the first year after AF diagnosis. Age-specific differences in stroke risk progression underscore the need for tailored reassessment intervals. Reassessment 6 months after AF diagnosis for patients aged ≥55 years and 1 year after AF diagnosis for those aged <55 years seems to be appropriate. Age accounted for most of the increased CHA_2_DS_2_-VA score, suggesting that routine reassessment at ages 65 and 75 should at least be performed. Regular risk reassessment is important for timely initiation of OACs and optimized management of comorbidities, potentially improving long-term outcomes.

## Supplementary Material

euaf145_Supplementary_Data

## Data Availability

Ethical and privacy reasons, as well as national health registry regulations, prohibit sharing of data. Further enquiries can be directed to the corresponding author.
