# Supplementary material for: Timing of stroke risk reassessment in atrial fibrillation patients with a CHA2DS2-VA score of 0 or 1: the Norwegian AFNOR study
Source: Europace. 2025 Jul 24;27(10):euaf145. doi: 10.1093/europace/euaf145 (PMC12509847; doi:10.1093/europace/euaf145)
Supplement: euaf145_Supplementary_Data [file euaf145_supplementary_data.docx]

**Supplementary material**

**Timing of stroke risk reassessment in atrial fibrillation patients with CHA_2_DS_2_**-**VA score of 0 or 1: the Norwegian AFNOR study**

Mariam Anjum^1,2,3^, Inger Ariansen^2^, Marius Myrstad^1,3^ Lars J. Kjerpeseth^2^, Vidar Hjellvik^2^, Eva Skovlund^5^, Ingrid E. Christophersen^1,6^, Arnljot Tveit^1,4^, Trygve Berge^1^.

1. Department of Medical Research, Bærum Hospital, Vestre Viken Hospital Trust, Gjettum, Norway
2. Department of Chronic Diseases, Norwegian Institute of Public Health, Oslo, Norway
3. Department of Internal Medicine, Bærum Hospital, Vestre Viken Hospital Trust, Gjettum, Norway
4. Department of Cardiology, Institute of Clinical Medicine, University of Oslo, Oslo, Norway
5. Department of Public Health and Nursing, Norwegian University of Science and Technology, Trondheim, Norway
6. Department of Medical Genetics, Oslo University Hospital, Oslo, Norway

The study was performed at Department of Medical Research, Bærum Hospital in Norway and
Department of Chronic Diseases, Norwegian Institute of Public Health, Oslo, Norway

**Mariam.anjum@vestreviken.no**

**Supplementary table of content:**

1. **Description of methodology:**

- Appendix A1: Definition of AF and exclusion criteria
- Appendix A2: Definition of CHA_2_DS_2_-VA score
- Supplementary Figure 1: Graphical representation of timeline for entry and exit

1. **Supplementary results:**

- Supplementary Table S1: Sex -stratified baseline characteristics
- Supplementary Table S2: Incidence rate of CHA_2_DS_2_-VA score increment
- Supplementary Table S3: Incidence rate of the CHA_2_DS_2_-VA risk factors stratified by sex
- Supplementary Table S4: Proportion of patients with an increase in the CHA_2_DS_2_-VA score and Number Needed to Reassess (NNR)
- Supplementary Table S5: Cumulative proportion with CHA_2_DS_2_-VA increment stratified by sex

__________________________________________________________________________________

**Appendix A1: Definition of AF and exclusion criteria**

| **Comorbidity** | **Norwegian Patient Registry** | **Norwegian Prescription Database** | | |
| --- | --- | --- | --- | --- |
|  | **ICD10 codes** | **ATC codes** | **Reimbursement codes** | |
|  |  |  | **ICD10 codes** | **ICPC2 codes** |
| *Atrial fibrillation | I48 | B01AA03 B01AE07 B01AF01 B01AF02 | I48 | K78 |
| Exclusion of patients with mitral stenosis | I05.0 I05.2 I34.2 Q23.2 | B01AC07 | I05.0 I05.2 I34.2 Q23.2 |  |
| Exclusion of patients with prosthetic heart valve | Z95.2 Z95.3 Z95.4 | B01AC07 | Z95.2 Z95.3 Z95.4 |  |

ICD10 = International Classification of Diseases, 10th revision. ICPC2 = International Classification of Primary Care, 2nd Edition. NPR = Norwegian Patient Registry. NorPD = Norwegian Prescription Database

**Appendix A2. Definition of the CHA_2_DS_2_VA score**

| **Comorbidity** | **Norwegian Patient Registry** | **Norwegian Prescription Database** | | |
| --- | --- | --- | --- | --- |
|  | **ICD10 codes** | **ATC codes** | **Reimbursement codes** | |
|  |  |  | **ICD10 codes** | **ICPC2 codes** |
| Congestive heart failure | I11.0 I42.0 I50  I13.0 I 13.2 | C01AA04 C01AA05 C01DA08 C01EB17 C02DB02 C03AA01 C03AA03 C03AB01 C03CA01 C03CA02 C03DA01 C03DA02 C03DA04 C03EA01 C07AA05 C07AB02 C07AB07 C07AG02 C09AA01 C09AA02 C09AA03 C09AA05 C09AA10 C09CA01 C09CA03 C09CA06 R03DA04 | I11.0 I42 I50 J81 | K77 |
| Hypertension | I10 I11 I12 I13 I15 O10 O11 | C02A C02C C02DB C02DC C03A C03CA01 C03CA04 C03DA01 C03DB01 C03E C07A C07B C08 C09  (÷ C07AA07 C08CA06) | I10 I11 I12 I13 I15 O10 O11 | K86 K87 |
| Diabetes mellitus | E10 E11 E12 E13 E14 O24.0 O24.1 O24.2 O24.3 | A10 |  |  |
| Ischemic stroke, transient ischemic attack, arterial thromboembolism | I63 I64 I74 G45 | B01AB04 B01AB05 B01AC04 B01AC05 B01AC07 B01AC30 | G45 I63 I74 | K89 K90 |
| Vascular disease | I21 I22 I23 I25.2 I70 I71 I72 I73.9 | B01AB01 B01AB04 B01AB05 B01AC04 B01AC07 B01AC22 B01AC24 B01AC26 C01DA02 C01DA08 C01DA14 C04AD03 C07AA05 C07AB02 C07AB03 C07AB07 C07AG01 C07AG02 C08CA01 C08CA02 C08CA05 C08DA01 C08DB01 C09AA01 C09AA03 C09AA05 C10 | -22 I21 I22 I70 I73.9 | -22 K75 K92 |
| *ICD-10 (International Classification of Diseases, 10th revision) were used in definitions of co-morbidities and outcomes. Comorbidities were recognized by ICD-10 diagnoses from hospital stays in the Norwegian Patient Registry (NPR). For typical primary care co-morbidities (heart failure, hypertension, diabetes and COPD) a drug dispensation in Norwegian Prescription Database (NorPD) was used as a proxy for an underlying condition. ATC (Anatomical Therapeutic Chemical system) codes from NorPD identified disease-specific drugs (e.g. anti-diabetics) and ICD-10 or International Classification for Primary Care 2 (ICPC-2) codes were used as reasons for reimbursement of drugs for chronic illnesses for less specific drugs (e.g. beta blockers). Heart failure, hypertension, vascular disease, and ischemic stroke, transient ischemic attack or systemic thromboembolism were defined as either an ICD-10 code from hospital stays or ATC code plus a reimbursement code on a dispensing. Diabetes mellitus was defined as an ICD-10 code from hospital stays or dispension of antidiabetic medication. Reimbursement code -22 refers to secondary prophylaxis after myocardial infarction in the Norwegian Blue Prescription scheme and is based on ICD-10 codes.* | | | | |

**Supplementary Figure 1:**

**
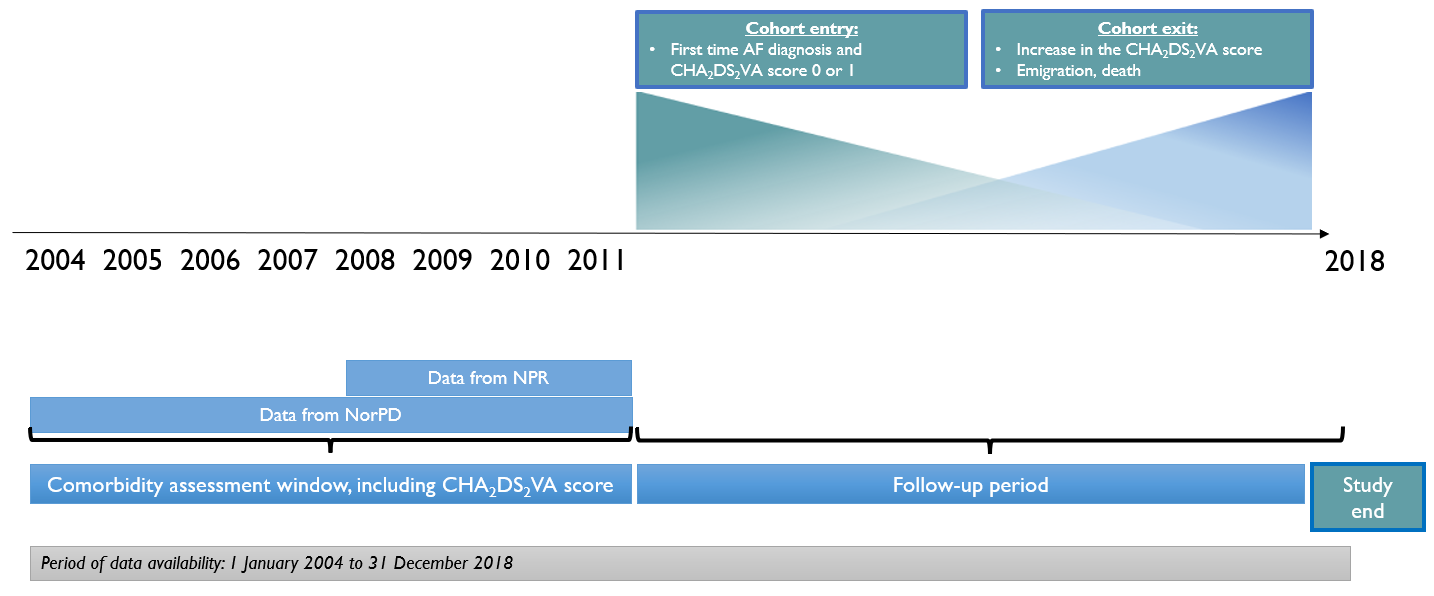
**

**Figure 1: Graphical representation of timeline for entry and exit:** The study population comprised Norwegian residents ≥ 18 years of age diagnosed with incident AF with a CHA_2_DD_2_-VA score of 0 or 1. Individuals were followed from date of registration of their first AF diagnosis after January 1^st^ 2011 (study start). Patients with AF diagnosis before study start and individuals with a diagnosis of prosthetic valve or mitral stenosis were excluded. We used a look-back period of minimum 3 years for assessment of AF and other comorbidities. Data from NPR is available from 2009 whereas data from NorPD is available from 2004. The CHA_2_DS_2_-VA score was scored based on diagnosis codes registered during the look-back period before study start and successively during follow-up. During the follow-up period, we continuously included individuals ≥18 years when recorded with their first AF diagnosis. Individuals were followed until an increase in the CHA_2_DS_2_-VA score, death, emigration or end of study (31.12.2018). The National Registry and Statistics Norway administrates data on all Norwegian residents with information on birth year, sex, emigration, changes in residency status and death. AF: Atrial Fibrillation, NPR: Norwegian patient registry, NorPD: Norwegian Prescription Database.

**Supplementary Table S1: Sex stratified baseline characteristics**

|  |  |  | |  |
| --- | --- | --- | --- | --- |
|  | **Low risk CHA_2_DS_2_-VA score 0** | | **Intermediate risk**  **CHA_2_DS_2_-VA score 1** | |
|  | **Men** | **Women** | **Men** | **Women** |
| n (%) | 11.985 (70.5) | 5.015 (29.5) | 15.328 (64.5) | 8.454 (35.5) |
| Mean age, years (SD) | 52.7 (9.7) | 54.0 (9.6) | 64.0 (8.4) | 66.1 (7.2) |
| Distribution of age, n (%) |  |  |  |  |
| Age 18-44 (%) | 2,539 (21.2) | 894 (17.8) | 519 (3.4) | 228 (2.7) |
| Age 45-54 (%) | 3,419 (28.5) | 1,251 (25.0) | 1,617 (10.6) | 520 (6.2) |
| Age 55-64 (%) | 6,027 (50.3) | 2,870 (57.2) | 4,852 (31.7) | 1,965 (23.2) |
| Age 65-74 (%) |  |  | 8,340 (54.4) | 5,741 (67.9) |
| CHA_2_DS_2_-VA risk factors |  |  |  |  |
| Heart failure (%) |  |  | 978 (6.4) | 309 (3.7) |
| Hypertension (%) |  |  | 4.271(27.9) | 1.902 (22.5) |
| Age 65-74 (%) |  |  | 8.340 (54.4) | 5.741 (67.9) |
| Diabetes (%) |  |  | 677 (4.4) | 256 (3.0) |
| Vascular disease (%) |  |  | 1.062 (6.9) | 246 (2.9) |
| HAS-BLED (SD) | 0.36 (0.6) | 0.40 (0.6) | 1.38 (0.7) | 1.41 (0.7) |
| Abnormal liver function (%) | 112 (0.9) | 90 (1.8) | 249 (1.6) | 173 (2.1) |
| Anemia (%) | 394 (3.3) | 361 (7.2) | 912 (6.0) | 730 (8.6) |
| Alcohol misuse (%) | 438 (3.7) | 108 (2.2) | 641 (4.2) | 165 (2.0) |
| Cancer (%) | 711 (5.9) | 439 (8.8) | 1.911 (12.5) | 1.119 (13.2) |
| Renal disease (%) | 223 (1.9) | 106 (2.1) | 757 (4.9) | 339 (4.0) |

**Supplementary results**

**Supplementary Table S2: Average annual incidence rate (per 100 person-years) of CHA_2_DS_2_-VA score increment**

|  | **All  Low + intermediate risk** | | **Low risk CHA_2_DS_2_-VA score 0** | **Intermediate risk**  **CHA_2_DS_2_-VA score 1** |
| --- | --- | --- | --- | --- |
|  | **IR (95% CI)** |  | **IR (95% CI)** | **IR (95% CI)** |
| **All** | 21.2(20.91-21.49) | | 15.84(15.48-16.21) | 26.17(25.72-26.62) |
| **Male** | 20.81(20.46-21.16) | | 15.68(15.25-16.11) | 26.02(25.47-26.58) |
| **Female** | 22.02(21.5-22.55) | | 16.24(15.57-16.93) | 26.44(25.69-27.21) |

**Supplementary Table S3: Average annual incidence rate (per 100 person-years) of the CHA_2_DS_2_-VA risk factors stratified by sex**

|  | **Low risk  CHA_2_DS_2_-VA score 0** | | **Intermediate risk  CHA_2_DS_2_-VA score 1** | |
| --- | --- | --- | --- | --- |
|  | **Male** | **Female** | **Male** | **Female** |
| **Hypertension** | 5.36(5.11- 5.61) | 4.94(4.57- 5.33) | 6.32(6.05- 6.6) | 7.19(6.81- 7.6) |
| **Age 65-74 years** | 5.72(5.47- 5.98) | 7.51(7.06- 7.99) | 5.13(4.89- 5.38) | 4.41(4.11- 4.74) |
| **Heart failure** | 2.02(1.87- 2.18) | 1.68(1.48- 1.92) | 3.6(3.4- 3.81) | 3.01(2.77- 3.28) |
| **Age >75 years** | **-** | **-** | 5.69(5.44- 5.96) | 7.91(7.51- 8.34) |
| **Vascular disease** | 1.23(1.11- 1.35) | 0.68(0.56- 0.84) | 2.65(2.48- 2.83) | 1.3(1.14- 1.48) |
| **Diabetes mellitus** | 0.62(0.54- 0.72) | 0.54(0.43- 0.68) | 1.11(1- 1.23) | 0.83(0.71- 0.98) |
| **Thromboembolism** | 1.1(0.99- 1.22) | 1.15(0.99- 1.35) | 2.09(1.94- 2.26) | 2.14(1.94- 2.37) |
| **Any** | 15.68(15.25- 16.11) | 16.24(15.57- 16.93) | 26.02(25.47- 26.58) | 26.44(25.69- 27.21) |

**Supplementary table S4: Proportion of patients with an increase in the CHA_2_DS_2_-VA score and Number Needed to Reassess (NNR) to find one patient with increased score at different time intervals after AF diagnosis:**

|  | **All  Low + intermediate risk** | | **Low risk  CHA_2_DS_2_-VA score 0** | | **Intermediate risk  CHA_2_DS_2_-VA score 1** | |
| --- | --- | --- | --- | --- | --- | --- |
|  | **Proportion (95% CI)** | **NNR (95% CI)** | **Proportion (95% CI)** | **NNR (95% CI)** | **Proportion (95% CI)** | **NNR (95% CI)** |
| ***4 months*** |  |  |  |  |  |  |
| **18-44** | 5,3 (4,6-6,0) | 19(17-22) | 5,0 (4,3-5,8) | 20 (17-23) | 6,6 (5,0-8,6) | 15 (12-20) |
| **45-54** | 9,7 (9,0-10,4) | 10 (10-11) | 10,1 (9,3-11,0) | 10 (9-11) | 8,8 (7,6-10,0) | 11 (10-13) |
| **55-64** | 18,2 (17.6-18.8) | 6 (5-6) | 18,9 (18,1-19,8) | 5 (5-6) | 17,1 (16,3-18,1) | 6 (6-6) |
| **65-74** | 19,1 (18,4-19,7) | 5 (5-5) |  |  | 19,1 (18,4-19,7) | 5 (5-5) |
| ***6 months*** |  |  |  |  |  |  |
| **18-44** | 6,3 (5,6- 7,1) | 16 (14-18) | 6,0 (5,3- 6,9) | 17 (15-19) | 7,5 (5,8- 9,6) | 13 (10-17) |
| **45-54** | 11,2 (10,5- 12,0) | 9 (8-10) | 11,7 (10,8- 12,6) | 9 (8-9) | 10,4 (9,1- 11,7) | 10 (9-11) |
| **55-64** | 22,0 (21,4- 22,7) | 5 (4-5) | 23,0 (22,1- 23,9) | 4 (4-5) | 20,7 (19,8- 21,7) | 5 (5-5) |
| **65-74** | 23,1 (22,4- 23,8) | 4 (4-4) |  |  | 23,1 (22,4- 23,8) | 4 (4-4) |
| ***1 years*** |  |  |  |  |  |  |
| **18-44** | 8,1 (7,3- 8,9) | 12 (11-14) | 7,6 (6,8- 8,6) | 13 (12-15) | 10,0 (8,1- 12,4) | 10 (8-12) |
| **45-54** | 14,1 (13,2- 14,9) | 7 (7-8) | 14,4 (13,5- 15,5) | 7 (6-7) | 13,2 (11,8- 14,7) | 8 (7-8) |
| **55-64** | 30,2 (29,5- 30,9) | 3 (3-3) | 30,8 (29,8- 31,8) | 3 (3-3) | 29,3 (28,3- 30,4) | 3 (3-4) |
| **65-74** | 30,4 (29,7- 31,2) | 3 (3-3) |  |  | 30,4 (29,7- 31,2) | 3 (3-3) |
| ***2 years*** |  |  |  |  |  |  |
| **18-44** | 10,2 (9,3- 11,2) | 10 (9-11) | 9,7 (8,7- 10,7) | 10 (9-11) | 12,7 (10,5- 15,3) | 8 (7-10) |
| **45-54** | 18,2 (17,3- 19,1) | 6 (5-6) | 18,8 (17,7- 19,9) | 5 (5-6) | 16,9 (15,4- 18,6) | 6 (5-7) |
| **55-64** | 41,6 (40,8- 42,4) | 2 (2-2) | 42,2 (41,2- 43,3) | 2 (2-2) | 40,8 (39,6- 42,0) | 2 (2-3) |
| **65-74** | 40,9 (40,1- 41,7) | 2 (2-2) |  |  | 40,9 (40,1- 41,7) | 2 (2-2) |
| ***3 years*** |  |  |  |  |  |  |
| **18-44** | 11,8 (10,8- 12,8) | 9 (8-9) | 11,2 (10,2- 12,3) | 9 (8-10) | 14,2 (11,9- 16,9) | 7 (6-8) |
| **45-54** | 20,7 (19,7- 21,6) | 5 (5-5) | 21,1 (19,9- 22,3) | 5 (4-5) | 19,8 (18,1- 21,5) | 5 (5-6) |
| **55-64** | 49,2 (48,4- 50,0) | 2 (2-2) | 49,8 (48,8- 50,9) | 2 (2-2) | 48,4 (47,2- 49,6) | 2 (2-2) |
| **65-74** | 47,8 (47,0- 48,6) | 2 (2-2) |  |  | 47,8 (47,0- 48,6) | 2 (2-2) |
| ***4 years*** |  |  |  |  |  |  |
| **18-44** | 13,0 (12,1- 14,1) | 8 (7-8) | 12,5 (11,4- 13,6) | 8 (7-9) | 15,8 (13,4- 18,6) | 6 (5-7) |
| **45-54** | 22,7 (21,7- 23,7) | 4 (4-5) | 23,2 (22,0- 24,4) | 4 (4-5) | 21,5 (19,8- 23,3) | 5 (4-5) |
| **55-64** | 54,6 (53,8- 55,4) | 2 (2-2) | 55,4 (54,4- 56,4) | 2 (2-2) | 53,5 (52,3- 54,7) | 2 (2-2) |
| **65-74** | 52,4 (51,6- 53,3) | 2 (2-2) |  |  | 52,4 (51,6- 53,3) | 2 (2-2) |
| ***5 years*** |  |  |  |  |  |  |
| **18-44** | 14,0 (13,0- 15,1) | 7 (7-8) | 13,3 (12,2- 14,5) | 8 (7-8) | 17,3 (14,7- 20,2) | 6 (5-7) |
| **45-54** | 24,2 (23,2- 25,2) | 4 (4-4) | 24,7 (23,5- 26,0) | 4 (4-4) | 23,0 (21,3- 24,8) | 4 (4-5) |
| **55-64** | 58,3 (57,6- 59,1) | 2 (2-2) | 59,2 (58,2- 60,2) | 2 (2-2) | 57,2 (56,0- 58,4) | 2 (2-2) |
| **65-74** | 55,6 (54,8- 56,5) | 2 (2-2) |  |  | 55,6 (54,8- 56,5) | 2 (2-2) |
| ***6 years*** |  |  |  |  |  |  |
| **18-44** | 14,7 (13,7- 15,9) | 7 (6-7) | 14,1 (12,9- 15,3) | 7 (7-8) | 17,9 (15,4- 20,9) | 6 (5-7) |
| **45-54** | 24,9 (23,9- 26,0) | 4 (4-4) | 25,5 (24,3- 26,8) | 4 (4-4) | 23,7 (21,9- 25,5) | 4 (4-5) |
| **55-64** | 60,6 (59,8- 61,4) | 2 (2-2) | 61,6 (60,6- 62,6) | 2 (2-2) | 59,2 (58,0- 60,4) | 2 (2-2) |
| **65-74** | 57,4 (56,5- 58,2) | 2 (2-2) |  |  | 57,4 (56,5- 58,2) | 2 (2-2) |
| ***7 years*** |  |  |  |  |  |  |
| **18-44** | 15,0 (14,0- 16,2) | 7 (6-7) | 14,4 (13,3- 15,6) | 7 (6-8) | 17,9 (15,4- 20,9) | 6 (5-7) |
| **45-54** | 25,4 (24,4- 26,4) | 4 (4-4) | 26,1 (24,9- 27,4) | 4 (4-4) | 23,8 (22,1- 25,7) | 4 (4-5) |
| **55-64** | 61,6 (60,9- 62,4) | 2 (2-2) | 62,6 (61,6- 63,6) | 2 (2-2) | 60,3 (59,1- 61,5) | 2 (2-2) |
| **65-74** | 58,5 (57,6- 59,3) | 2 (2-2) |  |  | 58,5 (57,6- 59,3) | 2 (2-2) |
| ***8 years*** |  |  |  |  |  |  |
| **18-44** | 15,3 (14,3- 16,5) | 7 (6-7) | 14,7 (13,5- 15,9) | 7 (6-7) | 18,3 (15,7- 21,3) | 5 (5-6) |
| **45-54** | 25,5 (24,5- 26,6) | 4 (4-4) | 26,3 (25,1- 27,6) | 4 (4-4) | 23,9 (22,1- 25,7) | 4 (4-5) |
| **55-64** | 61,9 (61,1- 62,7) | 2 (2-2) | 62,9 (61,9- 63,9) | 2 (2-2) | 60,6 (59,4- 61,7) | 2 (2-2) |
| **65-74** | 58,8 (58,0- 59,6) | 2 (2-2) |  |  | 58,8 (58,0- 59,6) | 2 (2-2) |

**Supplementary Table S5: Cumulative proportion with CHA_2_DS_2_-VA increment stratified by sex**

|  | **All  Low + intermediate risk** | | | **Low-risk  CHA_2_DS_2_-VA score 0** | | | **Intermediate-risk  CHA_2_DS_2_-VA score 1** | | |
| --- | --- | --- | --- | --- | --- | --- | --- | --- | --- |
|  | **All** | **Male** | **Female** | **All** | **Male** | **Female** | **All** | **Male** | **Female** |
| **4 months** | 15,7 (15.4-16.1) | 16,0 (15,6-16,5) | 15,2 (14,6-15,8) | 13,7 (13,2-14,2) | 13,9 (13,3-14,5) | 13,2 (12,2-13,2) | 17,2 (16,7-17,7) | 17,7 (17,1-18,3) | 16,3 (15,5-17,1) |
| **6 months** | 19,0 (18,6-19,4) | 19,2 (18,8-19,7) | 18,4 (17,8-19,1) | 16,5 (15,9-17,0) | 16,6 (16,0-17,3) | 16,1 (15,1-17,1) | 20,8 (20,3-21,3) | 21,3 (20,7-22,0) | 19,8 (19,0-20,7) |
| **1 year** | 25,3 (24,9-25,7) | 25,4 (24,9-25,9) | 25,1 (24,4-25,8) | 21,6 (21,0-22,3) | 21,6 (20,9-22,4) | 21,6 (20,5-22,7) | 27,9 (27,3-28,5) | 28,3 (27,6-29,1) | 27,2 (26,2-28,1) |
| **2 year** | 34,2 (33,7-34,7) | 34,1 (33,5-34,6) | 34,6 (33,8-35,4) | 29,2 (28,5-29,9) | 29,1 (28,3-29,9) | 29,4 (28,2-30,7) | 37,8 (37,2-38,4) | 37,9 (37,1-38,7) | 37,7 (36,7-38,7) |
| **3 year** | 40,1 (39,7-40,6) | 39,8 (39,2-40,4) | 40,8 (40,0-41,6) | 34,1 (33,4-34,9) | 33,9 (33,0-34,7) | 34,8 (33,5-36,1) | 44,4 (43,8-45,0) | 44,4 (43,6-45,2) | 44,4 (43,3-45,5) |
| **4 year** | 44,3 (43,7-44,7) | 39,8 (39,2-40,4) | 40,8 (40,0-41,6) | 37,9 (37,2-38,6) | 37,5 (36,7-38,4) | 38,7 (37,4-40,1) | 48,8 (48,2-49,4) | 48,7 (47,9-49,5) | 49,1 (48,0-50,1) |
| **5 year** | 47,2 (46,7-47,6) | 46,7 (46,1-47,3) | 48,1 (47,2-48,9) | 40,5 (39,7-41,2) | 40,3 (39,4-41,1) | 41,0 (39,6-42,4) | 51,9 (51,3-52,6) | 51,8 (51,0-52,6) | 52,2 (51,2-53,3) |
| **6 year** | 48,8 (48,3-49,3) | 48,4 (47,8-49,0) | 49,7 (48,8-50,5) | 42,1 (41,4-42,8) | 41,9 (41,0-42,8) | 42,6 (41,3-44,0) | 53,6 (53,0-54,3) | 53,5 (52,7-54,3) | 53,8 (52,8-54,9) |
| **7 year** | 49,7 (49,2-50,2) | 49,4 (48,8-50,0) | 50,4 (49,5-51,2) | 42,9 (42,2-43,6) | 42,7 (41,8-43,6) | 43,3 (41,9-44,6) | 54,6 (54,0-55,2) | 54,6 (53,8-55,4) | 54,6 (53,5-55,6) |
| **8 year** | 50,0 (49,5-50,5) | 49,7 (49,1-50,3) | 50,7 (49,8-51,5) | 43,1 (42,4-43,9) | 43,0 (42,1-43,9) | 43,5 (42,1-44,0) | 54,9 (54,3-55,5) | 54,9 (54,1-55,7) | 54,9 (53,9-56,0) |
